# 5-Nitrotetrazol and 1,2,4-Oxadiazole Methylene-Bridged Energetic Compounds: Synthesis, Crystal Structures and Performances

**DOI:** 10.3390/molecules26237072

**Published:** 2021-11-23

**Authors:** Jiarong Zhang, Fuqiang Bi, Zhi Yang, Qi Xue, Bozhou Wang

**Affiliations:** 1 Xi’an Modern Chemistry Research Institute, Xi’an 710065, China; sonia610@126.com (J.Z.); xueq704@163.com (Q.X.); 2 School of Chemistry and Chemical Engineering, Beijing Institute of Technology, Beijing 100081, China; zhiyang@bit.edu.cn; 3 State Key Laoratory of Fluorine & Nitrogen Chemicals, Xi’an 710065, China

**Keywords:** nitrotetrazole, 1,2,4-oxadiazole, methylene-bridged energetic compound, synthesis, performances

## Abstract

A new structural type for melt cast materials was designed by linking nitrotetrazole ring with 1,2,4-oxadiazole through a N-CH_2_-C bridge for the first time. Three N-CH_2_-C linkage bridged energetic compounds, including 3-((5-nitro-2H-tetrazol-2-yl) methyl)-1,2,4-oxadiazole (NTOM), 3-((5-nitro-2H-tetrazol-2-yl)methyl)-5-(trifluoromethyl)-1,2,4 -oxadiazole (NTOF) and 3-((5-nitro-2H-tetrazol-2-yl)methyl)-5-amine-1,2,4-oxadiazole (NTOA), were designed and synthesized through a two-step reaction by using 2-(5-nitro-2H-tetrazole -2-yl)acetonitrile as the starting material. The synthesized compounds were fully characterized by NMR (^1^H, ^13^C), IR spectroscopy and elemental analysis. The single crystals of NTOM, NTOF and NTOA were successfully obtained and investigated by single-crystal X-ray diffraction. The thermal stabilities of these compounds were evaluated by DSC-TG measurements, and their apparent activation energies were calculated by Kissinger and Ozawa methods. The crystal densities of the three compounds were between 1.66 g/cm^3^ (NTOA) and 1.87 g/cm^3^ (NTOF). The impact and friction sensitivities were measured by standard BAM fall-hammer techniques, and their detonation performances were computed using the EXPLO 5 (v. 6.04) program. The detonation velocities of the three compounds are between 7271 m/s (NTOF) and 7909 m/s (NTOM). The impact sensitivities are >40 J, and the friction sensitivities are >360 N. NTOM, NTOF and NTOA are thermally stable, with decomposition points > 240 °C. The melting points of NTOM and NTOF are 82.6 °C and 71.7 °C, respectively. Hence, they possess potential to be used as melt cast materials with good thermal stabilities and better detonation performances than TNT.

## 1. Introduction

Melt cast explosives are widely used in military and civilian fields such as grenades, mortars, warheads and antipersonnel mines [1]. Although the melting points of melt cast explosives are generally between 70 and 120 °C, a compound possessing melting point below 100 °C is ideal in order to use steam for low-cost melting operations [2]. 2,4,6-Trinitrotoluene (TNT) and dinitroanisole (DNAN) are two kinds of widely investigated and applied melt cast materials. However, their further applications are limited by their relative shortcomings, such as the toxicity and environmental problems of TNT [3,4] and low detonation performances of DNAN [5,6]. Therefore, there is continued interest in developing new melt cast materials with better explosive performances.

**Figure 1:**
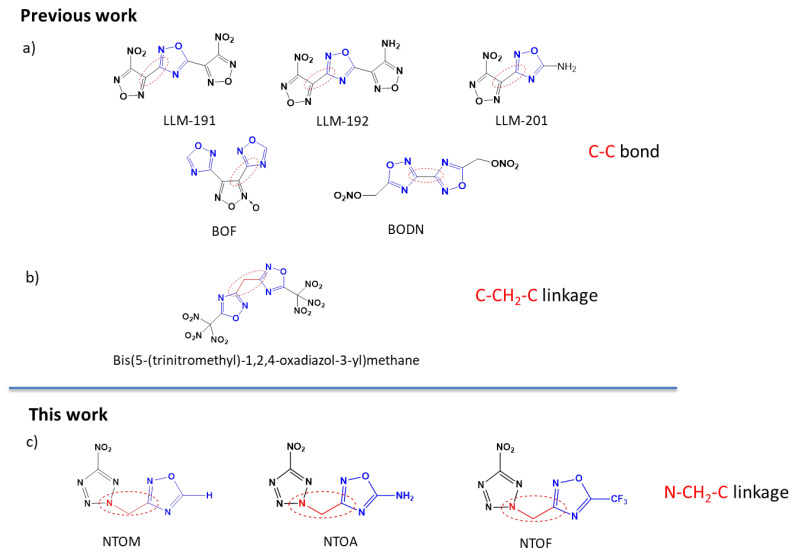
(**a**) Previous 1,2,4-oxadiazole energetic materials with C-C bond linkage; (**b**) methylene bridged 1,2,4-oxadiazole melt cast material with C-CH_2_-C linkage; (**c**) methylene bridged 1,2,4-oxadiazole melt cast material with N-CH_2_-C linkage.

In the past decade, significant progress has been made in the field of melt cast explosives. Researchers have found that the 1,2,4-oxadiazole skeleton is promising for the construction of new high explosives with balanced detonation and safety properties [7,8,9,10]. A lot of melt cast energetic materials have been synthesized based on 1,2,4-oxadiazole, such as 3,5-bis(4-nitrofurazan-3-yl)-1,2,4-oxadiazole (LLM-191) [11], 3-(4-amino-1,2,5-oxadiazol-3-yl)-5-(4-nitro-1,2,5-oxadiazol-3-yl)-1,2,4-oxadiazole (LLM-192) [11], 3-(4-nitro-1,2,5-oxadiazol-3-yl)-1,2,4-oxadiazol-5-amine (LLM-201) [12], bis(1,2,4-oxadiazolyl)furoxan (BOF) [13] and bis(1,2,4-oxadiazole)bis(methylene) dinitrate (BODN) [14] (Figure 1a). In recent years, several design strategies on the structures and properties of melt cast explosives have been developed. For example, the combination of 1,2,4-oxadiazoles with other heterocyclic rings bearing energetic groups that exhibit melt-castable characteristics, the introduction of bridged alkyl chains in heterocycles and the incorporation of fluorine or trifluoromethyl groups on heterocyclic rings are effective methods for the construction of highly energetic melt cast materials [15]. Recently, Tian Lu [16] et al. reported a low melting point compound bis(5-(trinitromethyl)-1,2,4-oxadiazol-3-yl)methane (*T*_m_: 52 °C), which is composed of two 1,2,4-oxadizole rings bridged by C-CH_2_-C linkage (Figure 1b). Although bis(5-(trinitromethyl)-1,2,4-oxadiazol-3-yl)methane possesses good detonation performances (*D*: 9053 m/s, *P*: 37.4 GPa), its mechanical sensitivities are relatively high (*IS*: 12.5 J, *FS*: 72 N), and the decomposition point is only 117 °C. Hence, this compound is not thermally stable, and its wide application is limited.

Herein, for the first time, we designed a new kind of structure by linking 1,2,4-oxadiazole and nitrotetrazole ring via N-CH_2_-C linkage. We expect that this could be a promising new strategy for constructing energetic compounds that possess good detonation parameters, better thermal stabilities and lower sensitivity toward impact and friction. In 2019, we firstly synthesized NTOA [17]_,_ but detailed structural characteristics and performance investigations were not investigated. Herein, based on our previous studies, two new energetic compounds (NTOF and NTOM) were firstly designed and synthesized (Figure 1c). All of the synthesized compounds in this study were characterized by nuclear magnetic resonance (NMR) and infrared (IR) spectroscopy. X-ray crystallographic measurements of the three compounds were performed, providing insight into structural characteristics as well as intramolecular and intermolecular interactions of these compounds. The thermal stabilities were evaluated by DSC-TG measurements, and the detonation and sensitivity performances were investigated comprehensively.

## 2. Results and Discussions

### 2.1. Synthesis

**Scheme 1:**
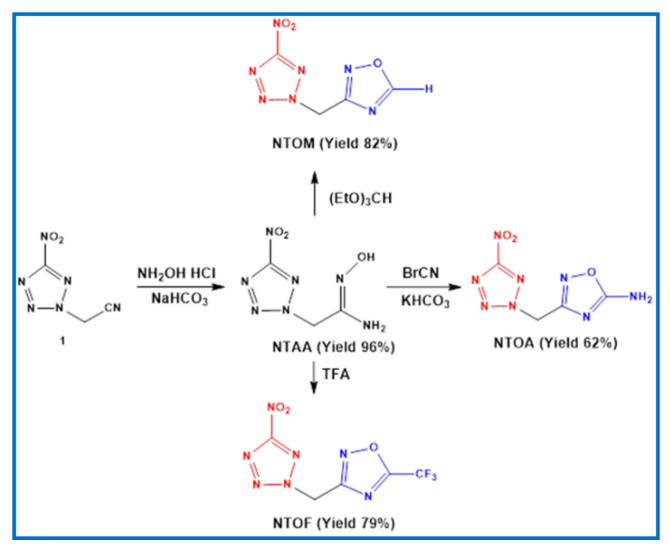
The synthesis approaches of NTOM, NTOF and NTOA.

5-nitrotetrazol-3-acetonitrile (1) was prepared according to the method we developed previously [17]. The intermediate compound N’-hydroxy-2-(5-nitro-2H-tetrazol-2-yl)acetimidamide (NTAA) was synthesized from compound one in the presence of hydroxylamine hydrochloride with a yield of 96%. Based on NTAA, three methyl-bridged nitrotetrazole and 1,2,4-isofurazan compounds including NTOM, NTOF and NTOA were synthesized with yields of 82%, 79% and 62%, respectively. In order to synthesize energetic compounds with better energy performances, we tried to oxidize and nitrate the amino group of NTOA to the nitro or nitramine group, but failed to obtain the targeted compounds (Scheme 1).

### 2.2. X-ray Crystallography

**Figure 2:**
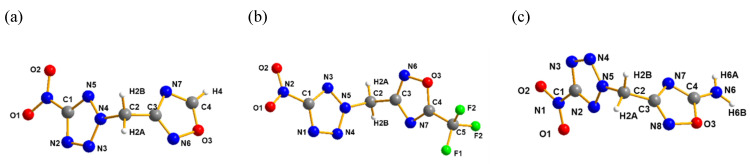
Single-crystal X-ray structures of NTOM (**a**), NTOF (**b**) and NTOA (**c**).

The crystal structures of NTOM, NTOF and NTOA were obtained and analysed by X-ray single crystal diffraction (Figure 2). The crystal data and structure refinement parameters were given in Tables S1–S4 in the SI.

NTOM, NTOF and NTOA crystalized in the monoclinic system and contained four molecules in a unit cell. The space group of NTOM, NTOF and NTOA are P(1)2(1)/n(1), P2(1)/c and P2(1)/n, respectively. The crystal density of NTOM is 1.76 g∙cm^−3^ at 296(2) K. After introduction of -CF_3_, crystal density was obviously improved to 1.87 g∙cm^−3^. However, the introduction of -NH_2_ into NTOM causes crystal density of NTOA to decrease to 1.66 g∙cm^−3^. As illustrated in Figure 2c, the amino group and 1,2,4-oxadiazole are in the same plane, and the atoms in 5-nitrotetrazole are essentially planar with very small torsion angle (−1.76°) of N3-C1-N1-O2. Meanwhile, the C-N bond length and the N-N bond length in oxadiazole and tetrazole rings are found to be shorter than the normal C-N single bond (1.47 Å) and longer than the C = N double bond (1.22 Å). These results indicate that electronic conjugation is formed in amino-1,2,4-oxadiazole and 5-nitrotetrazole structure.

**Figure 3:**
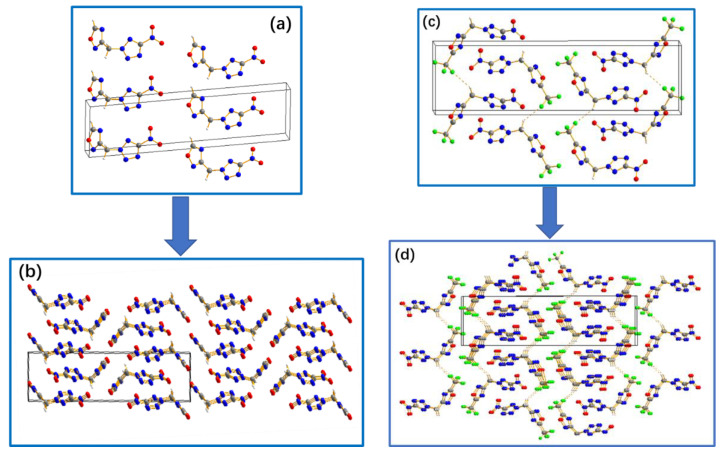
(**a**) Two-dimensional image of NTOM; (**b**) stacking diagram of NTOM; (**c**) two-dimensional image of NTOF; (**d**) stacking diagram of NTOF.

**Figure 4:**
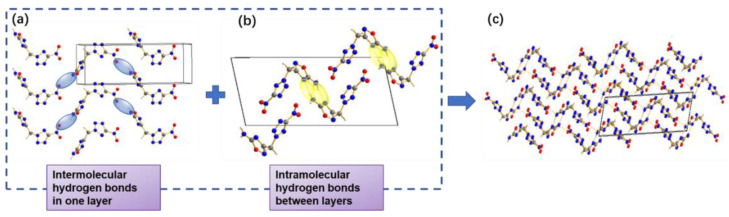
(**a**) Intermolecular hydrogen bonds in one layer of NTOA; (**b**) intramolecular hydrogen bonds between adjacent layers of NTOA; (**c**) stacking diagram of NTOA.

From Figure 3a, there are no typical hydrogen bonds formed in NTOM, and each NTOM molecule exists independently. The stacking diagram (Figure 3b) indicated that two adjacent NTOM molecules in one layer were arranged in reverse and the NTOM molecules in different layers occluded with each other; thus, the molecular arrangement is relatively compact. In NTOF (Figure 3c,d), the hydrogen bond C2-H2A…F1 causes the molecules between two adjacent layers to be more closely arranged. Meanwhile, due to the introduction of fluorine atoms, the density of NTOM further increased, in comparison with NTOM. Unlike NTOM and NTOF, there are two kinds of typical hydrogen bonds in NTOA. The hand-in-hand intermolecular hydrogen bond N6-H6B…O1 (Figure 4a) constructs the 2D layer structure of NTOA, and the adjacent layers are connected by the face-to-face hydrogen bond N6-H6A…N7 (Figure 4b). The length of both the two hydrogen bonds in NTOA is shorter than 3.0 Å, which are within the range of strong hydrogen bonds. The presence of a large number of hydrogen bonds is beneficial for improving the density and stability of NTOA. Due to the –CH2- group connection, the dihedral angle of N7-C3-C2-N5 is 49.71°, and the stacked structure of NTOA is polyline-like (Figure 4c).

### 2.3. Thermal Stabilities

**Figure 5:**
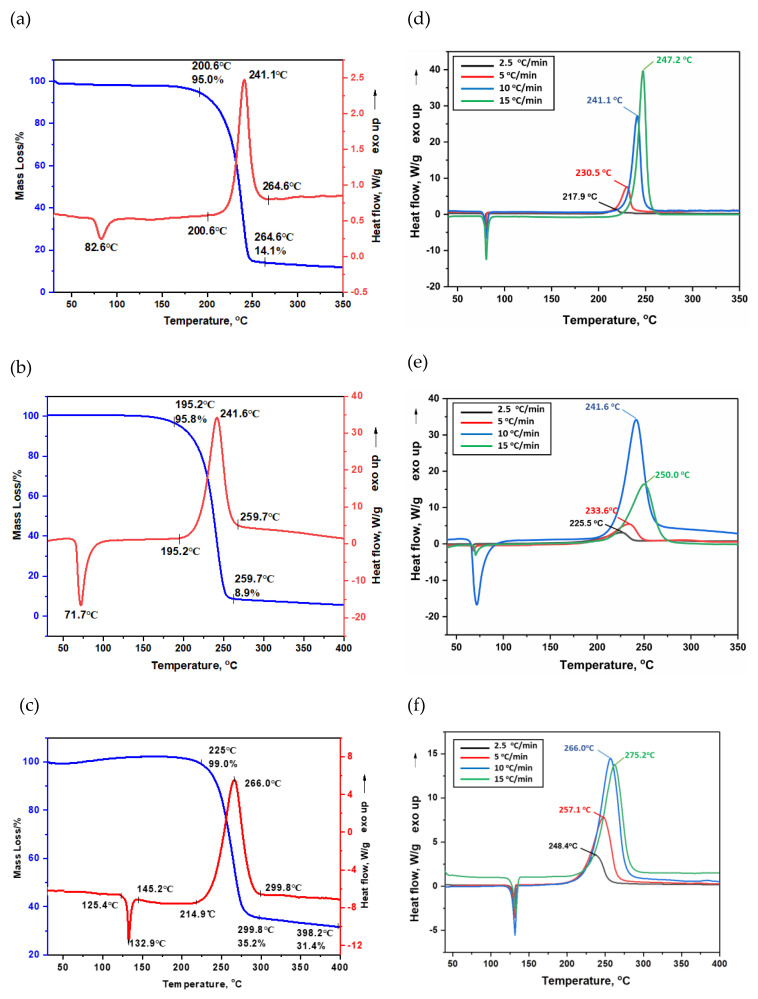
The DSC-TG traces of NTOM (**a**), NTOF (**b**) and NTOA (**c**); the DSC traces of NTOM (**d**), NTOF (**e**) and NTOA (**f**) obtained at different heating rates.

Figure 5a–c show the DSC-TG traces of NTOM, NTOF and NTOA under a heating rate of 10 °C min^−1^. All of the three energetic materials are thermal stable with decomposition points of above 240 °C. NTOM melts at 82.6 °C and decomposes at 241.1 °C, suggesting its great potential to be used as melt cast explosive. Compared with NTOM, the melting point of NTOF reduced to 71.7 °C, and the decomposition point improved to 241.6 °C due to the introduction of the -CF_3_ group. With the help of the abundant inter and intra hydrogen bonds formed in NTOA, the melting point (132.9 °C) and decomposition point (266.0 °C) both increased in comparison with NTOM. In general, the thermal stabilities evaluated from DSC can be summarized as NTOA (266.0 °C) > NTOF (241.6 °C) > NTOM (241.1 °C).

In order to obtain further information about the thermal decomposition processes of NTOM, NTOA and NTOF, the reaction kinetic parameters during the heating process were studied by Kissinger’s [18] and Ozawa–Doyle’s method [19]. The DSC curves at different hating rates (2.5, 5, 10 and 15 °C min^−1^) were shown in Figure 5. The apparent activation energy E for the thermal decomposition of NTOM calculated by Kissinger and Ozawa method is 121.54 kJ mol^−1^ and 121.56 kJ mol^−1^, respectively (Table 1). After the introduction of -CF_3_ and -NH_2_, the apparent activation energies for the thermal decomposition of NTOF and NTOA were both increased. Notably, the linear correlation coefficients (r) calculated by Kissinger’s method and Ozawa’s method of the three compounds are very close and greater than 0.98. Thus, the calculated results are credible and provide a good reference for the thermal safety of NTOM, NTOA and NTOF.

### 2.4. Sensitivities

The sensitivities of NTOM, NTOF and NTOA toward impact and friction were tested using BAM methods, and the results are listed in Table 2. The three compounds possess satisfactory sensitivities with the IS of > 40 J and FS > 360 N.

The impact sensitivity of energetic materials are very closely related to their electrostatic potential (ESP) [20,21,22,23,24,25]. We calculated the ESP of NTOM, NTOF and NTOA using the Gaussian 09 method at the theoretical level of B3LYP/6-311g (d, p) before the sensitivity test.

**Figure 6:**
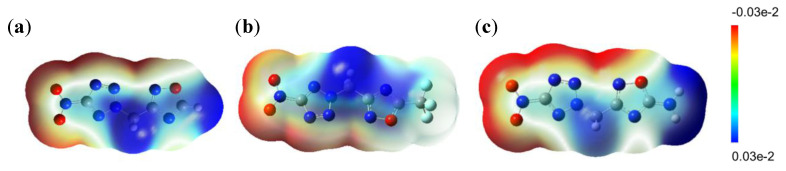
Electrostatic potential of NTOM (**a**), NTOF (**b**) and NTOA (**c**) (B3LYP//6-311g(d, p), 0.001 electron/b3 isosurface, energy values –0.03 to + 0.03 H). Color coding: yellow, slightly negative; green, neutral; turquoise, slightly positive; light blue, positive; dark blue, very positive.

It can be observed from Figure 6 that the negative charges of the three compounds are mainly distributed around nitrotetrazole, while the positive charges are concentrated on the N-CH_2_-C linkage. Positive charge is distributed on the amino group connected with isofurazan in the NTOA compound and the hydrogen atom connected with isofurazan in NTOM. The trifluoromethyl group in NTOF is distributed with partial positive charge. In most N-O compound systems, especially those containing nitro groups, the concentration of more positive charges around N atoms will result in an imbalance, which will theoretically result in higher impact sensitivity. The N atoms of the three compounds have no obvious positive charge distribution. Therefore, we believe that the sensitivities of the three compounds are low. 

### 2.5. Hirshfeld Analysis

**Figure 7:**
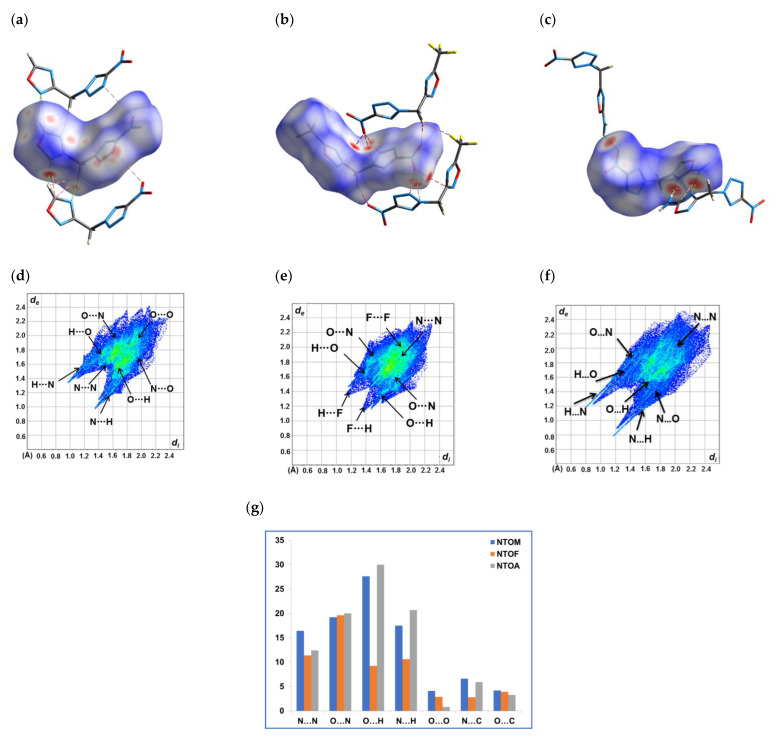
Hirshfeld surface of NTOM (**a**); Hirshfeld surface of NTOF (**b**); 2D fingerprint plot of NTOA (**c**); 2D fingerprint plot of NTOM (**d**); 2D fingerprint plot of NTOF (**e**); 2D fingerprint plot of NTOA (**f**); comparison of the amounts of close interactions in NTOM, NTOF and NTOA (**g**).

The Hirshfeld surfaces (*d*_norm_) of NTOM, NTOF and NTOA were calculated by CrystalExplorer [26] (Figure 7a−c). The 3D *d*_norm_ surface was used to identify close intermolecular interactions of compounds. The blue and red regions represent closer and longer contacts, respectively, and the white regions mean the distance of contacts equal to the v_dW_ separation with a *d*_norm_ value of zero. The green dotted lines represent the hydrogen bonds formed in the molecule, and the red dotted lines are close contacts.

The red dots appeared on the surfaces of NTOM and NTOA are mainly resulted from intermolecular hydrogen bonds formed in the compounds, while the red areas on the surfaces of NTOF are caused by close contacts of the compound. The red dots on NTOM and NTOA attributed to HBs are dark, which indicates the presence of strong hydrogen bonds. As observed from Figure 7a−c, the three compounds have relatively few “hot spots,” indicating that their sensitivities to stimuli are relatively low.

The 2D fingerprint plots clearly illustrate the different contributions of intermolecular interactions (Figure 7d−f), and the comparison of the amounts of close interactions in NTOM, NTOF and NTOA are shown in Figure 7g. It has been reported that the O…H and N…H interactions tend to decrease sensitivities, while the O…O interactions usually increase the sensitivity of a compound [27]. The percentage of contribution of O∙∙∙H, N∙∙∙H, N∙∙∙O and N∙∙∙N type interactions in the total Hirshfeld surface of NTOM are 27.6%, 17.5%, 19.2% and 16.4%, respectively. These values are similar to the results of NTOA (the percentage of contribution of O∙∙∙H, N∙∙∙H, N∙∙∙O and N∙∙∙N type interactions in the total Hirshfeld surface of NTOA is 30%, 20.7%, 20% and 12.4%, respectively). As there are only two H atoms exist in NTOF, the percentage of contribution of O∙∙∙H and N∙∙∙H interactions are only 9.2% and 10.6%. The percentage of N∙∙∙O and N∙∙∙N type interactions of NTOF are 19.6% and 11.4%, respectively. The N∙∙∙C and O∙∙∙C type of interactions contribute asmall amount in the total surface of the three compounds. The O∙∙∙O contact interactions of NTOM, NTOA and NTOF are only 4.1%, 0.8% and 2.9%, which indicate that the three compounds are insensitive to external mechanical stimuli. The Hirshfeld analysis is in good consistency with the ESP analysis and experiment results.

### 2.6. Physiochemical Properties and Detonation Performances of NTOM, NTOF and NTOA

The physiochemical and energetic properties of NTOM, NTOF and NTOA compared with TNT and BODN are summarized in Table 2. Density is one of the most important factors for evaluating the detonation performances of energetic compounds [28,29]. The crystal densities of NTOM, NTOF and NTOA were 1.66, 1.76 and 1.87 g/cm^3^, respectively. The introduction of the -CF_3_ group makes the density of NTOF apparently higher than that of NTOM, while the introduction of -NH_2_ makes the density of NTOA lower than that of NTOM. Moreover, the densities of these three compounds are higher than that of TNT (1.65 g/cm^3^). The solid-state enthalpies of formation of NTOM and NTOA are positive and obtained as 275.8 kJ/mol and 344.9 kJ/mol, respectively. NTOM possesses a detonation velocity of 7909 m/s and detonation pressure of 24.80 Gpa. The detonation velocity of NTOA is 7451 m/s, and the detonation pressure is 29.3 GPa. However, the solid-state enthalpy of formation of NTOF is negative (−328.8 kJ/mol), which renders the detonation pressure and detonation velocity of NTOF lower than those of NTOM and NTOA.

The sensitivities of the three compounds to impact and friction were tested by BAM drop hammer and BAM friction tester. The impact sensitivities of NTOM, NTOA and NTOF are >40 J and the friction sensitivities are >360 N. In comparison with TNT, NTOM and NTOF have better detonation performances, thermal stabilities and sensitivities. In terms of detonation performance, BODN has more outstanding detonation velocity (*D*: 8180 m/s) and detonation pressure (*P*: 29.4 GPa) than those of NTOM and NTOF. The thermal decomposition temperatures of NTOM and NTOF are greater than 240 °C, which are much higher than that of BODN (*T*_d_: 183.4 °C). Meanwhile, the impact sensitivities of NTOM and NTOF are apparently lower than that of BODN (*IS*: 8.7 J, *FS*: 282 N). 

## 3. Experiments

General caution! Although we have experienced no explosion accidents in synthesis and characterization of these materials, proper protective measures should be adopted.

### 3.1. Materials and Measurements

2-(5-Nitro-2H-tetrazol-2-yl)acetonitrile (1) was prepared according to the literature [17]. Hydroxylamine hydrochloride, sodium bicarbonate, methanol, potassium bicarbonate, cyanogen bromide, trichloroacetic acid (TFA), tetrahydrofuran (THF), triethyl orthoformate((EtO)_3_CH) and BF_3_·Et_2_O were commercially available and used without further purification. ^1^H NMR and ^13^C NMR of NTAA, NTOA, NTOM and NTOF were recorded on 500 MHz (Bruker AVANCE 500) nuclear magnetic resonance spectrometers. The melting and decomposition points were determined using a differential scanning calorimeter (TA Instruments Company, Model DSC–Q200) at a flow rate of N_2_ at 50 mL min^−1^. About 0.3 mg of the sample was sealed in aluminium pans for DSC analysis. Infrared spectra were obtained from KBr pellets on a Nicolet NEXUS870 Infrared spectrometer in the range of 4000~400 cm^−1^. Elemental analyses (C, H and N) were performed on a VARI–El–3 elementary analysis instrument. The impact and friction sensitivities were determined by using the BAM method.

### 3.2. X-ray Crystallography

The diffraction data of NTOA, NTOM and NTOF were collected on a BRUKER SMART Apex II CCD X-ray diffractometer equipped with a Mo Kα radiation (λ = 0.71073 A) using the *ω-θ* scan mode. The structures were solved by the direct method using SHELXS-97 and refined with full-matrix least-squares procedures on F2 with SHELXL-97. The crystal data and structure refinement parameters were listed in Table S1 (see Supplementary Materials). The selected bond lengths, bond angles and hydrogen bond data were summarized in Tables S2–S7. The crystal structures of NTOM, NTOF and NTOA have been deposited with the Cambridge Crystallographic Data Centre (CCDC), under deposition numbers 2,114,990, 2,114,991 and 1,947,762. These data can be obtained free of charge from The Cambridge Crystallographic Data Centre via https://www.ccdc.cam.ac.uk/ (accessed on 11 October 2021).

### 3.3. Synthesis of NTAA

Compound 1 (0.46 g, 3.0 mmol), hydroxylamine hydrochloride (0.32 g, 4.6 mmol) and NaHCO_3_ (0.39 g, 4.6 mmol) were added to MeOH (5 mL). After refluxing the reaction solution for 2 h, the inorganic salt was removed by filtration, and the solvent was evaporated to provide a yellow solid NTAA (0.54 g, 96%). ^1^H NMR (500 MHz, DMSO-*d*_6_): δ ppm: 5.460 (s, 2H, CH), 5.853 (s, 2H, NH), 9.640 (s, 1H, OH); ^13^C NMR (125 MHz, DMSO-*d*_6_): δ ppm: 55.103, 146.499,166.254; IR ν: 3485, 3382, 2871, 2813, 1678, 1598, 1555, 1476, 1420, 1325, 910, 844, 775, 705, 659 cm^−1^; elemental analysis calculated (%) for compound 2: C 19.54, H 2.72, N 52.44; found: C19.26, H 2.69, N 52.40.

### 3.4. Synthesis of NTOA

NTAA (0.3 g, 1.6 mmol) was added to a solution of KHCO_3_ (0.53 g, 5.3 mmol) in water (10 mL). The reaction solution was heated to clear (45 ℃), and cooled to room temperature. BrCN (0.26 g, 2.4 mmol) was added portionwise. The reaction solution was stirred overnight; then, the light brown solid was formed and filtered. The filter cake was washed with a small amount of cold water and diethyl ether and dried naturally to provide the product NTOA (0.2 g, 62%). ^1^H NMR (500 MHz, DMSO-*d*_6_):δ ppm: 8.093 (s, 2H, NH), 6.169 (s, 2H, CH); ^13^C NMR (125 MHz, DMSO-*d*_6_): δ ppm: 173.022, 166.540, 164.323, 50.176; IR ν: 3421, 3361, 3139, 3020, 2979, 1678, 1565, 1484, 1429, 1341, 1321, 848, 757, 634 cm^−1^; elemental analysis calculated (%) for compound NTOA: C 22.65, H 1.90, N 52.82; found: C 22.60, H 1.92, N 53.01.

### 3.5. Synthesis of NTOM

Triethyl orthoformate (3.96 g, 26.7 mmol) and NTAA (0.5 g, 2.67 mmol) were added into three-necked flask, then BF_3_·Et_2_O was added dropwise at room temperature. After 4 h, the reaction solution was poured into ice, white solid was precipated and filtered. Washed with small amount of cold water, 0.43 g NTOM was obtained with a yield of 82%. ^13^C NMR (125 MHz, CDCl_3_-*d*_6_): δ ppm: 49.36 (CH_2_), 162.42, 166.45 (tetrazol and oxadiazole circle); ^1^H NMR (500 MHz, CDCl_3_-*d*_6_): δ ppm: 6.16 (s, CH2), 8.85(s, CH); IR ν: 3129, 3022, 2975, 1559, 1483, 1419, 1322, 1279, 1103, 1030, 848, 618 cm^−1^; elemental analysis calculated (%) for compound NTOM: C 24.37, H 1.53, N 49.74; found: C 24.41, H 1.51, N 49.70.

### 3.6. Synthesis of NTOF

NTAA (0.72 g, 3.85 mmol) was added to dry THF (4 mL), then TFAA (2.4 g, 11.55 mmol) was added dropwise. The solution was heated to reflux for 24 h. Twenty-five percent aqueous ammonia solution was added to neutralize the reaction solution to pH = 7–8. Saturated NaCl aqueous solution was added, and ethyl acetate was used for extraction. The solvent evaporated, and 0.8 g NTOF was obtained with a yield of 79%. ^13^C NMR (500 MHz, CDCl_3_-*d*_6_): δ ppm: 49.17 (CH_2_), 115.01 (-CF_3_),164.10, 167.80 (tetrazol and oxadiazole circle); ^1^H NMR (500 MHz, CDCl_3_-*d*_6_): δ ppm: 6.23 (s, CH_2_); ^19^F NMR (470.5 MHz, CDCl_3_-*d*_6_): δ ppm: −65.24 (-CF_3_); IR ν: 3448, 3047, 2986, 2878, 1609, 1568, 1482, 1347, 1184, 1449, 995, 846, 773, 653 cm^−1^; elemental analysis calculated (%) for compound NTOF: C 22.65, H 0.76; found: C 22.50, H 0.77.

## 4. Conclusions

In this study, a new structural type for melt cast materials was designed by linking, for the first time, a nitrotetrazole ring with 1,2,4-oxadiazole through a N-CH_2_-C bridge. Three N-CH_2_-C-bridged energetic compounds, namely NTOM, NTOF and NTOA, were synthesized starting from 2-(5-nitro-2H-tetrazole-2-yl)acetonitrile. The three compounds were fully characterized by NMR, IR spectroscopy and elemental analysis. In addition, the single crystals of NTOM, NTOF and NTOA were successfully obtained and investigated by using single-crystal X-ray diffraction. Crystal densities of the three compounds are in the range of 1.66 g/cm^3^ (NTOA)~1.87 g/cm^3^ (NTOF). Calculated detonation velocities are between 7271 m/s (NTOF) and 7909 m/s (NTOM). The impact sensitivities are >40 J, and the friction sensitivities are >360 N. NTOM, NTOF and NTOA are thermally stable with decomposition points above 240 °C. These compounds possess good thermal stabilities. The melting points of NTOM and NTOF are 82.6 °C and 71.7 °C, respectively. Hence, these compounds hold the potential to be used as melt cast materials with better performances than TNT. This study demonstrates that the construction of N-CH_2_-C linkage in heterocycles is an effective strategy to reduce sensitivity and improve thermal stabilities of energetic materials.

## Figures and Tables

**Table 1:** Kinetic parameters and enthalpies of thermal decomposition of NTOA, NTOM and NTOF.

	*β* (K·min^−1^)	*T*_p_ (K)	E/(kJ·mol^−1^)		r		lg (A_k_/s^−1^)
			Kissinger	Ozawa	Kissinger	Ozawa	Kissinger
NTOA	2.5	521.55	152.72	153.68	0.98549	0.9871	14.55
	5	530.25					
	10	539.15					
	15	548.35					
NTOM	2.5	491.05	121.54	123.56	0.99565	0.99613	12.2
	5	503.65					
	10	514.25					
	15	520.35					
NTOF	2.5	498.65	153.05	153.61	0.98583	0.98734	15.32
	5	506.75					
	10	514.75					
	15	523.15					

Notes: E is the apparent activation energy; A_k_ is pre-exponential factor; r is the liner correlation coefficient.

**Table 2:** Physicochemical properties and detonation performances of NTOA, NTOM, NTOF, TNT and BODN.

Compounds	NTOA	NTOM	NTOF	TNT	BODN [14]
ρ ^(a)^ (g/cm^3^)	1.66	1.76	1.87	1.65	1.83
Δ_f_*H* ^(b)^ (kJ/mol)	275.8	344.9	−328.8	−67	−79.4
T_m_ ^(c)^ (℃)	131.6	82.6	71.7	81	84.5
T_d_ ^(d)^ (℃)	256.6	241.1	261.6	295	183.4
Ω ^(e)^ (%)	−60.67	−0.53	−0.48	−74	−33.3
D ^(f)^ (m/s)	7451	7909	7271	6881	8180
*P*^(g)^ (GPa)	20.89	24.80	23.66	19.5	29.4
IS ^(h)^ (J)	>40	>40	>40	15	8.7
FS ^(i)^ (N)	>360	>360	>360	240	282

^(a^^)^ Density. ^(b)^ Heats of formation calculated by Gaussian 09. ^(c)^ Melting temperature (endothermic peak). ^(d)^ Decomposition temperature at 10 ℃/min (exothermic peak). ^(e)^ Oxygen balance (based on CO_2_) for C_a_H_b_O_c_N_d_, 16(c − (2a + 0.5b))/MW, MW = molecular weight. ^(f)^ Calculated detonation velocity (EXPLO5 v 6.04). ^(g)^ Calculated detonation pressure (EXPLO5 v 6.04). ^(h)^ Impact sensitivity evaluated by a standard BAM fall-hammer. ^(i)^ Friction sensitivity evaluated by BAM technique.

## Data Availability

Not applicable.

## Supplementary Materials

The following are available online, Table S1: Crystal data and structure refinement parameters for NTOM, NTOF and NTOA, Tables S2–S4: Selected bond lengths (Å) and bond angles (°) of NTOM, NTOF and NTOA, Tables S5–S7: Hydrogen bond lengths (Å) and bong angles (°) of NTOM, NTOF and NTOA, Figure S1: ^1^H NMR spectrum of NTAA, Figure S2: ^13^CNMR spectrum of NTAA, Figure S3:^1^H NMR spectrum of NTOA, Figure S4: ^13^C NMR spectrum of NTOA, Figure S5: ^1^H NMR spectrum of NTOM, Figure S6: ^13^C NMR spectrum of NTOM, Figure S7:^1^H NMR spectrum of NTOF, Figure S8:^13^C NMR spectrum of NTOF, Figure S9: ^19^F NMR spectrum of NTOF, Figures S10–S12: IR spectrum of NTOA, NTOM, NTOF.

## Sample Availability

Samples of the compounds are available from the authors.
