# Supplementary material for: 5-Nitrotetrazol and 1,2,4-Oxadiazole Methylene-Bridged Energetic Compounds: Synthesis, Crystal Structures and Performances
Source: Molecules. 2021 Nov 23;26(23):7072. doi: 10.3390/molecules26237072 (PMC8658944; doi:10.3390/molecules26237072)
Supplement: Supplementary file 1 [file molecules-26-07072-s001.zip › Supporting information.pdf]

Supporting Information

5-Nitrotetrazol and 1,2,4-oxadiazole Methylene-bridged Energetic Compounds: Synthesis, Crystal structures and Performances

Jiarong Zhang [1,2], Fuqiang Bi [1,3*], Zhi Yang [2], Qi Xue[1,3], and Bozhou Wang [1,3*]

[1] Xi'an Modern Chemistry Research Institute, Xi'an 710065, People's Republic of China; sonia610@126.com

[2] School of Chemistry and Chemical Engineering, Beijing Institute of Technology, Beijing 100081, China

[3] State Key Laboratory of Fluorine & Nitrogen Chemicals, Xi'an 710065, People's Republic of China;

* Correspondence: wbz600@163.com; Tel: +86-029-88294197

Table of contents

Table S1. Crystal data and structure refinement parameters for NTOM, NTOF and NTOA

Table S2. Selected bond lengths (Å) and bond angles (°) of NTOM

Table S3. Selected bond lengths (Å) and bong angles (°) of NTOF

Table S4. Selected bond lengths (Å) and bong angles (°) of NTOA

Table S5.Hydrogen bond lengths (Å) and bong angles (°) of NTOM

Table S6.Hydrogen bond lengths (Å) and bong angles (°) of NTOF

Table S7.Hydrogen bond lengths (Å) and bong angles (°) of NTOA

Figure S1.^{1}H NMR spectrum of NTAA.

Figure S2. ^{13}CNMR spectrum of NTAA.

Figure S3.^{1}H NMR spectrum of NTOA.

Figure S4. ^{13}C NMR spectrum of NTOA.

Figure S5. ^{1}H NMR spectrum of NTOM.

Figure S6. ^{13}C NMR spectrum of NTOM.

Figure S7.^{1}H NMR spectrum of NTOF.

Figure S8.^{13}C NMR spectrum of NTOF.

Figure S9. ^{19}F NMR spectrum of NTOF.

Figure S10.IR spectrum of NTOA

Figure S11. IR spectrum of NTOM.

Figure S12.IR spectrum of NTOF.

Crystallographic data

The apparatus and conditions of crystal structure determination

A single crystal of NTOM suitable for X-ray diffraction analysis was prepared by slow evaporation of ethyl acetate solvent at room temperature. A colorless crystal suitable for X-ray single crystal diffraction analysis were obtained by slow evaporation ofethyl acetate solvent at room temperature. The diffraction data were collected on a BRUKER SMART Apex II CCD X-ray diffractometer equipped with a Mo *K*α radiation (λ=0.71073 A) using an ω-θ scan mode at 150(2) K. A total of 5242 reflections were obtained in the range of 6.28≤θ≤54.99°, of which 1398 were independent (*R* int =0.0377) were observed and used for the refinement. The structure was solved by direct methods and refined by full-matrix least-squares techniques on F^2 using SHELES-97 and SHELXL-97 programs. A full-matrix least-squares refinement gave the final R_1= 0.0383 and ωR_2= 0.1085 (ω=1/[$\sigma^2(F_0^2)$ + $(0.0270\ P)^2$ + 0.0000 *P*], where $P = (F_0^2+2F_c^2)/3$). The goodness-of-fit on F^2 is 1.030. The largest difference peak and hole were 0.238 and -0.313 e/Å^3

A single crystal of NTOF suitable for X-ray diffraction analysis was prepared by slow evaporation of ethyl acetate/petroleum ether solvent at room temperature. The diffraction data were collected on a BRUKER SMART Apex II CCD X-ray diffractometer equipped with a Mo *K*α radiation (λ=0.71073 A) using an ω-θ scan mode at 296(2) K. A total of 2809 reflections were obtained in the range of 2.27≤θ≤25.10°, of which 1959 were independent (*R* int =0.0181) were considered to be observed and used for the refinement. The structure was solved by direct methods and refined by full-matrix least-squares techniques on F^2 using SHELES-97 and SHELXL-97 programs. A full-matrix least-squares refinement gave the final R_1=

0.061 and ωR_2= 0.1797 (ω=1/[$\sigma^2(F_0^2)$ + (0.0270 $P)^2$ + 0.0000 P], where P =(F_0^2+2F_c^2)/3). The goodness-of-fit on F^2 is 1.035. The largest difference peak and hole were 0.555 and -0.394 e/Å^3.

A single crystal of NTOA suitable for X-ray diffraction analysis was prepared by slow evaporation of ethyl acetate/petroleum ether solvent at room temperature. The diffraction data were collected on a BRUKER SMART Apex II CCD X-ray diffractometer equipped with a Mo $K\alpha$ radiation (λ=0.71073 A) using an ω-θ scan mode at 296(2) K. A total of 4031 reflections were obtained in the range of 2.33≤θ≤25.10° , of which 1507 were independent (R int =0.0217) were considered to be observed and used for the refinement. The structure was solved by direct methods and refined by full-matrix least-squares techniques on F^2 using SHELES-97 and SHELXL-97 programs. A full-matrix least-squares refinement gave the final R_1= 0.0375 and ωR_2= 0.0850 (ω=1/[$\sigma^2(F_0^2)$ + (0.0270 $P)^2$ + 0.0000 P], where P =(F_0^2+2F_c^2)/3). The goodness-of-fit on F^2 is 1.056. The largest difference peak and hole were 0.158 and -0.130 e/Å^3.

Table S1. Crystal data and structure refinement parameters for NTOM, NTOF and NTOA

Compound	NTOM	NTOF	NTOA
Empirical formula	$C_4H_3N_7O_3$	$C_5H_2F_3N_7O_3$	C4H4N8O3
Molar mass (g/mol)	197.13	265.14	212.15
Temperature (K)	150(2)	150(2)	296(2)
Crystal system	Monoclinic	Monoclinic	Monoclinic
Space group	*P*121/n1	*P*2(1)/c	*P*2(1)/n
a (Å)	5.4256(14)	4.9309(4)	7.9677(16)
b (Å)	6.4011(13)	7.3376(5)	6.1015(12)

c (Å)	21.522(4)	26.010(2)	17.905(3)
α (°)	90	90	90
β (°)	95.564(13)	92.560(3)	102.565(3)
γ (°)	90	90	90
V (Å^3)	743.9(3)	940.1(1)	849.6(3)
Z	4	4	4
h	-4<= *h*<=6	-5<= *h*<=6	-7<= *h*<=9
k	-7<= *k*<=7	-9<= *k*<=7	-7<= *k*<=6
l	-26<= *l*<=26	-32<= *l*<=32	-21<= *k*<=18
*D*c (g/cm^3)	1.760	1.873	1.659
λ (Å)	0.71073	0.71073	0.71073
μ (Mo K) (mm^{-1})	0.849	0.191	0.143
F(0 0 0)	400	528	432
θ range (°)	6.28-54.99	5.77-52.768	2.33-25.10
Measured reflections	5242	5516	4031
Unique data (*R*int)	1398 (0.0377)	1914 (0.0327)	1507 (0.0217)
*R*1, *wR*2 [$I > 2\sigma(I)$]	0.0383,0.1085	0.0390, 0.0799	0.0375, 0.0850
*R*1, *wR*2 (all data)	0.0404, 0.1116	0.0578, 0.0913	0.0375, 0.0850
Goodness-of-fit	1.030	1.095	1.056
δpmax, δpmin (e/ Å^3)	0.238, -0.313	0.26, -0.22	0.158, -0.130
CCDC number	2114990	2114991	1947762

Table S2. Selected bond lengths (Å) and bond angles (°) of NTOM

Bond	Dist.	Bond	Dist.
O(1)-N(1)	1.2222(16)	C(1)-N(5)	1.3193(18)
C(1)-N(2)	1.3334(19)	C(1)-N(1)	1.4430(18)
N(1)-O(2)	1.2191(16)	C(2)-N(4)	1.4571(18)
C(2)-C(3)	1.488(2)	C(2)-H(2A)	0.99
C(2)-H2AB	0.99	N(2)-N(3)	1.3126(18)
O(3)-C(4)	1.3211(19)	O(3)-N(6)	1.3964(17)
C(3)-N(6)	1.3047(19)	C(3)-N(7)	1.3726(19)
N(3)-N(4)	1.3271(17)	N(4)-N(5)	1.3226(16)
C(4)-N(7)	1.305(2)	C(4)-H(4)	0.95
Angle	(°)	Angle	(°)

N5-C1-N2	115.24(13)	N5-C1-N1	122.51(12)
N2-C1-N1	122.25(13)	O(1)-N(2)-C(1)	118.36(10)
O(1)-N(2)-C(2)	120.35(10)	O2-N1-O1	125.42(13))
O2-N1-C1	117.56(12)	O1-N1-C1	117.01(12)
N4-C2-C3	110.15(11)	N4-C2-H2A	109.6
C3-C2-H2A	109.6	N4-C2-H2AB	109.6
C3-C2-H2AB	109.6	H2A-C2-H2AB	108.1
N3-N2-C1	104.79(12)	C4-O3-N6	106.92(11)
N6-C3-N7	114.84(13)	N6-C3-C2	121.59(13)
N7-C3-C2	123.55(13)	N2-N3-N4	105.80(11)
N5-N4-N3	114.81(11)	N5-N4-C2	123.81(12)
N3-N4-C2	121.38(11)	N7-C4-O3	113.69(13)
N7-C4-H4	123.2	O3-C4-H4	123.2
C1-N5-N4	99.36(11)	C4-N7-C3	101.55(12)
C3-N6-O3	103.00(11)		

Table S3. Selected bond lengths (Å) and bong angles (°) of NTOF

Bond	Dist.	Bond	Dist.
F(1)-C(5)	1.315(2)	N(3)-N(5)	1.324(2)
F(2)-C(5)	1.326(2)	N(3)-C(1)	1.315(2)
F(3)-C(5)	1.314(2)	N(4)-N(5)	1.324(2)
O(1)-N(2)	1.220(2)	N(5)-C(2)	1.460(2)
O(2)-N(2)	1.2207(19)	N(6)-C(3)	1.301(3)
O(3)-N(6)	1.401(2)	N(7)-C(3)	1.378(2)
O(3)-C(4)	1.336(2)	N(7)-C(4)	1.280(2)
N(1)-C(1)	1.333(2)	C(4)-C(5)	1.499(3)
Angle	(°)	Angle	(°)
C4-O3-N6	105.15(14)	N3-C1-N1	122.30(16)
N4-N1-C1	104.28(15)	N5-C2-C3	110.94(15)
O1-N2-O2	125.81(17)	N6-C3-N7	115.18(18)
O1-N2-C1	117.57(15)	N6-C3-C2	120.98(17)
O2-N2-C1	116.61(16)	N7-C3-C2	123.84(17)
C1-N3-N5	99.73(14)	O3-C4-C5	117.00(18)

N1-N4-N5	106.19(14)	N7-C4-O3	115.36(17)
N3-N5-C2	123.15(15)	N7-C4-C5	12758(18)
N7-N5-N3	114.37(14)	F1-C5-F2	107.72(18)
N4-N5-C2	122.48(14)	F1-C5-C4	110.96(17)
C3-N6-O3	103.14(15)	F2-C5-C4	110.46(17)
C4-N7-C3	101.16(16)	F3-C5-F1	108.64(17)
N2-C2-N2	122.19(16)	F3-C5-F2	107.28(18)
N3-C1-N1	115.42(16)	F3-C5-C4	111.62(18)

Table S4. Selected bond lengths (Å) and bong angles (°) of NTOA

Bond	Dist.	Bond	Dist.
N(1)-O(2)	1.1997(19)	N(1)-O(1)	1.2162(18)
N(1)-C(1)	1.447(2)	N(2)-C(1)	1.310(2)
N(2)-N(5)	1.3146(17)	N(3)-N(4)	1.316(2)
N(3)-C(1)	1.326(2)	N(4)-N(5)	1.3196(18)
N(5)-C(2)	1.453(2)	N(6)-C(4)	1.310(2)
N(6)-H(6A)	0.8600	N(6)-H(6B)	0.8600
N(7)-C(4)	1.3079(19)	N(7)-C(3)	1.3549(18)
N(8)-C(3)	1.2876(19)	N(8)-O(3)	1.4289(17)
O(3)-C(4)	1.3444(17)	C(2)-C(3)	1.488(2)
C(2)-H(2A)	0.9700	C(2)-H(2B)	0.9700
Angle	(°)	Angle	(°)
O(2)-N(1)-O(1)	125.89(15)	O(2)-N(1)-C(1)	117.67(15)
O(1)-N(1)-C(1)	116.44(14)	C(1)-N(2)-N(5)	100.05(13)
N(4)-N(3)-C(1)	104.29(13)	N(3)-N(4)-N(5)	106.18(13)
N(2)-N(5)-N(4)	114.20(13)	N(2)-N(5)-C(2)	123.47(13)
N(4)-N(5)-C(2)	122.33(13)	C(4)-N(6)-H(6A)	120.0
C(4)-N(6)-H(6B)	120.0	H(6A)-N(6)-H(6B)	120.0
C(4)-N(7)-C(3)	102.04(12)	C(3)-N(8)-O(3)	102.42(11)
C(4)-O(3)-N(8)	105.71(11)	N(2)-C(1)-N(3)	115.28(14)
N(2)-C(1)-N(1)	122.82(14)	N(3)-C(1)-N(1)	121.88(14)
N(5)-C(2)-C(3)	110.18(12)	N(5)-C(2)-H(2A)	109.6

C(3)-C(2)-H(2A)	109.6	N(5)-C(2)-H(2B)	109.6
C(3)-C(2)-H(2B)	109.6	H(2A)-C(2)-H(2B)	108.1
N(8)-C(3)-N(7)	116.76(13)	N(8)-C(3)-C(2)	120.68(13)
N(7)-C(3)-C(2)	122.54(13)	N(7)-C(4)-N(6)	128.67(14)
N(7)-C(4)-O(3)	113.06(13)	N(6)-C(4)-O(3)	118.26(13)

Table S5.Hydrogen bond lengths (Å) and bong angles (°) of NTOM

D-H…A	d(D-H) (Å)	d(H…A) (Å)	d(D…A) (Å)	∠DHA(°)
C2-H2A…N7	0.99	2.58	3.511(2)	156.3
C4-H4…O3	0.95	2.59	3.2570(19)	127.7
C4-H4…N6	0.95	2.43	3.251(2)	144.4

Table S6.Hydrogen bond lengths (Å) and bong angles (°) of NTOF

D-H…A	d(D-H) (Å)	d(H…A) (Å)	d(D…A) (Å)	∠DHA(°)
C2—H2A…F1	0.860	2.264	2.959	137.93

Table S7.Hydrogen bond lengths (Å) and bong angles (°) of NTOA

D-H…A	d(D-H) (Å)	d(H…A) (Å)	d(D…A) (Å)	∠DHA(°)
N6-H6A…N7	0.860	2.115	2.966	169.72
N6-H6B…O1	0.860	2.223	2.948	141.96

NMR spectra

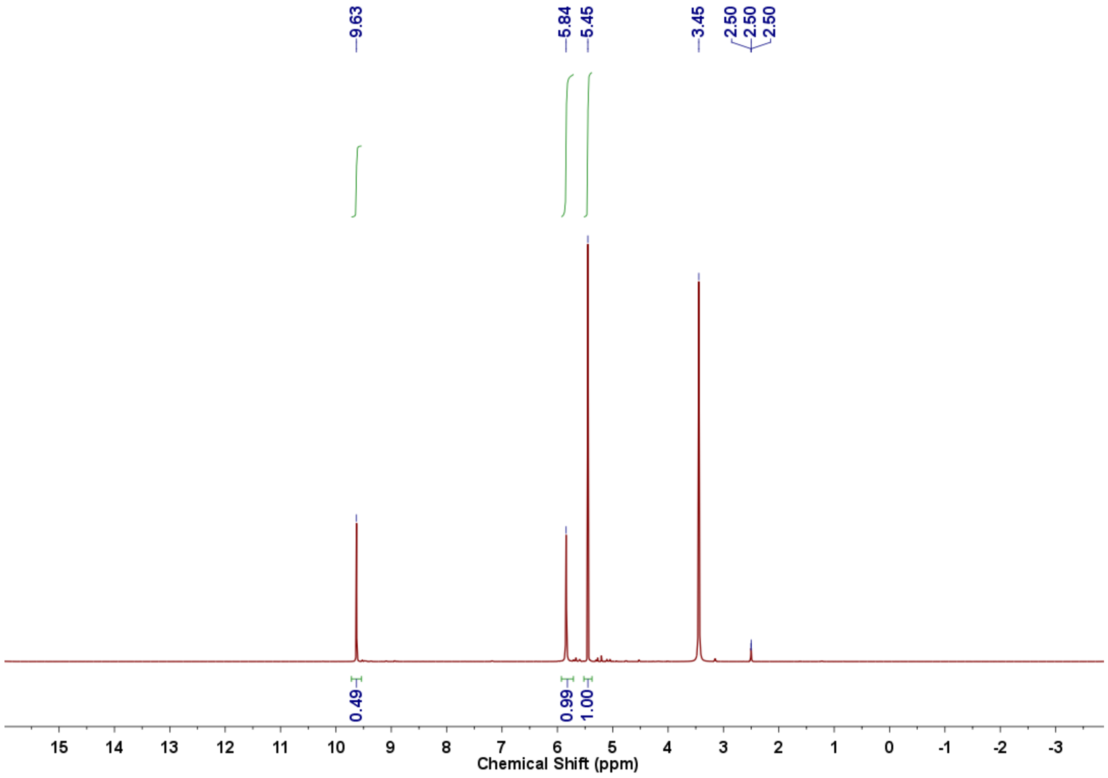

Figure S1. ^{1}H NMR spectrum of NTAA.

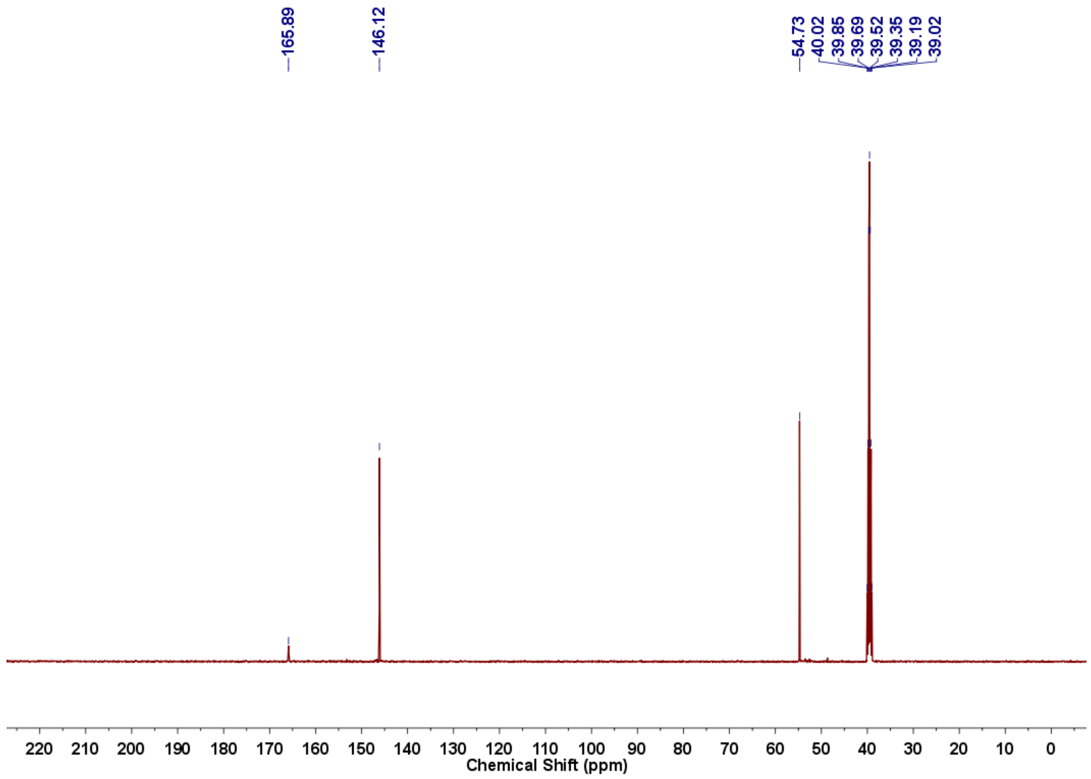

Figure S2. ^{13}CNMR spectrum of NTAA.

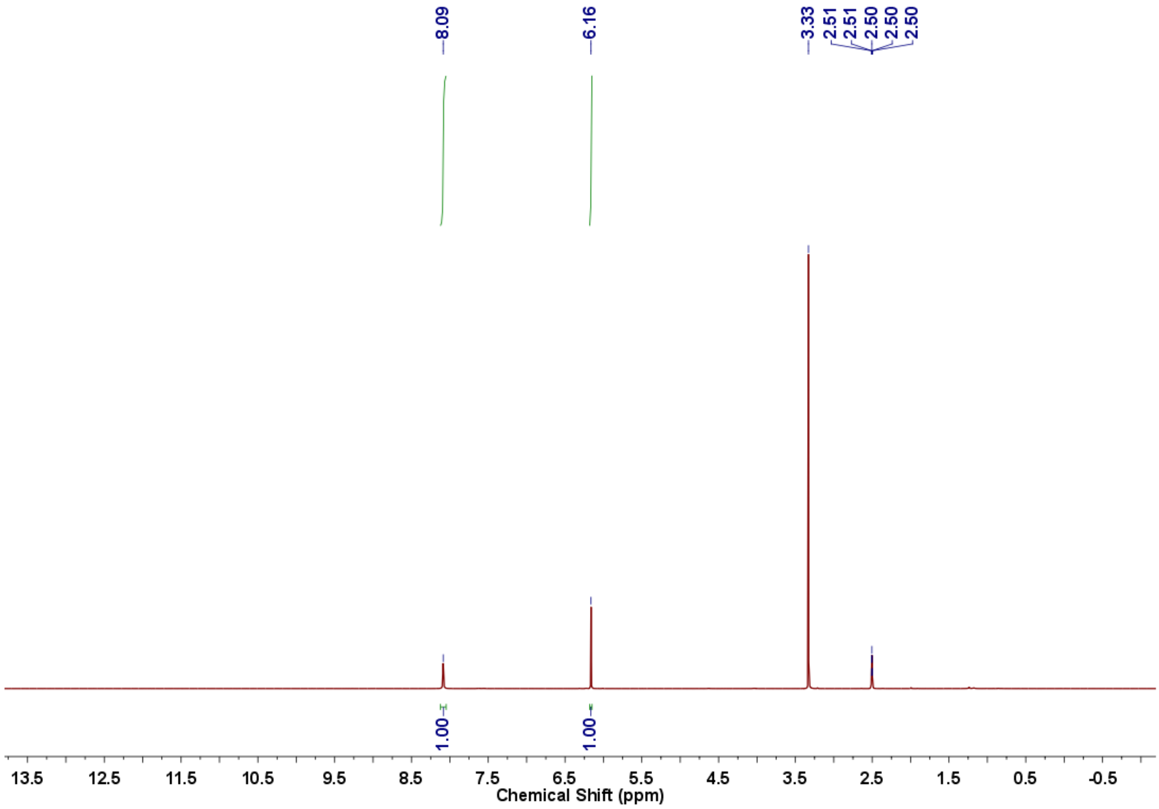

Figure S3. ^{1}H NMR spectrum of NTOA.

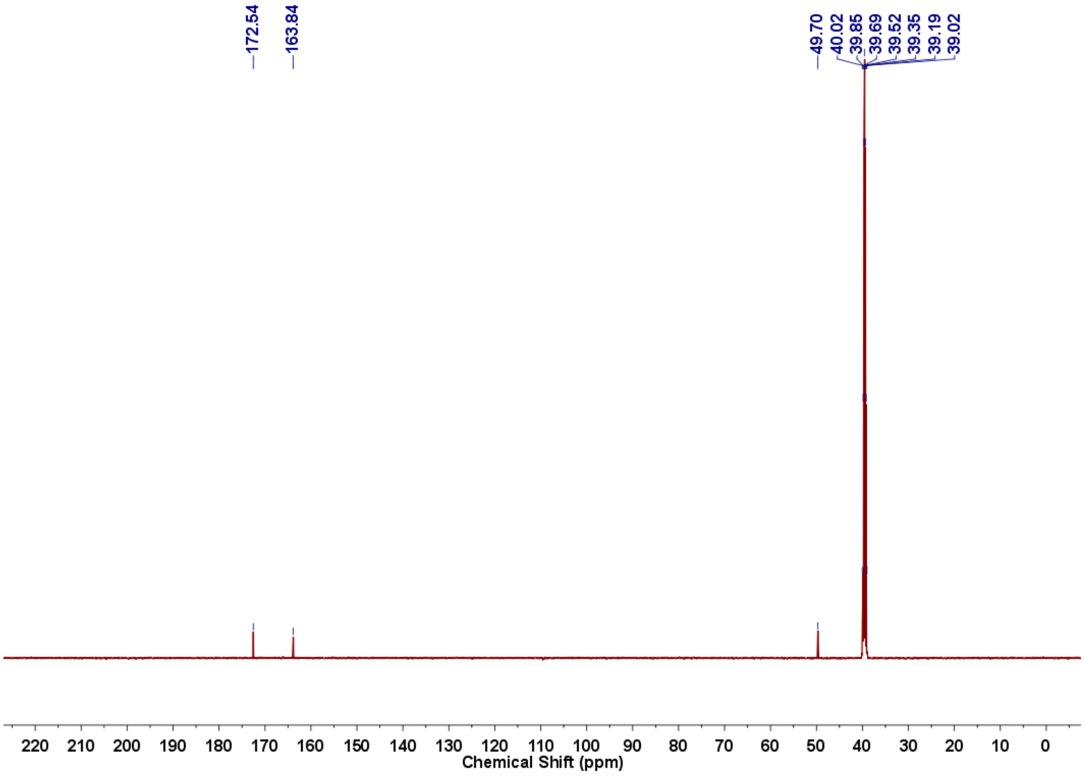

Figure S4. ^{13}C NMR spectrum of NTOA.

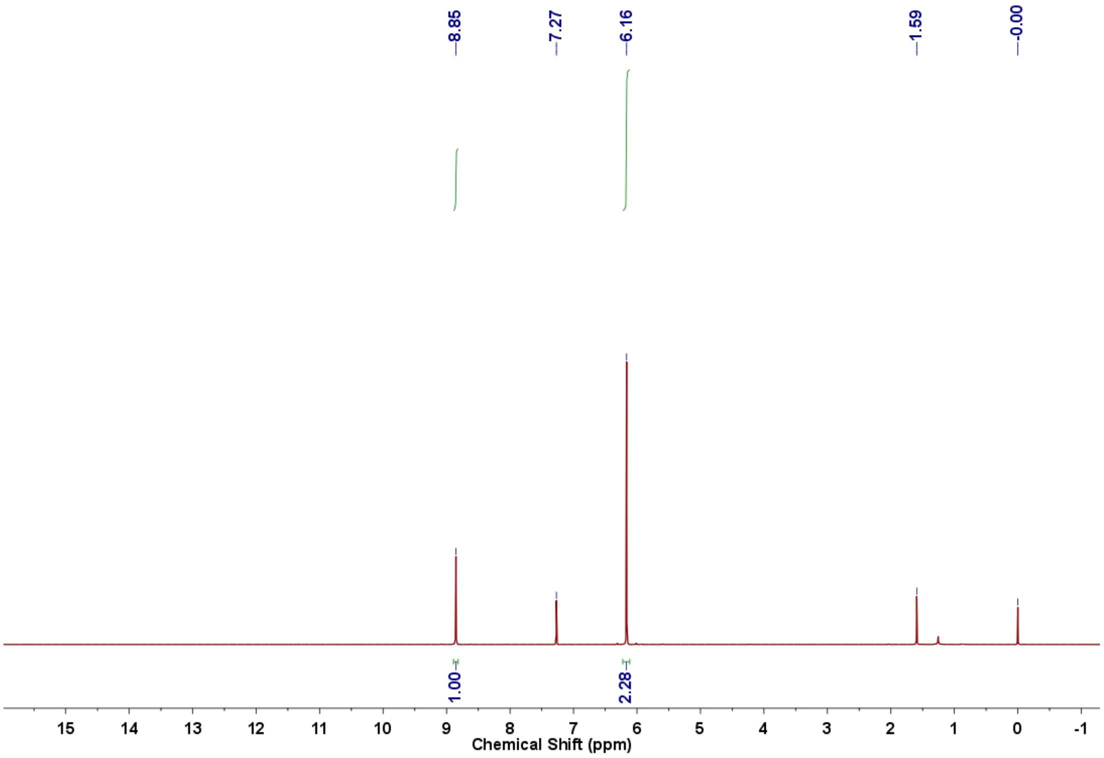

Figure S5. ^{1}H NMR spectrum of NTOM.

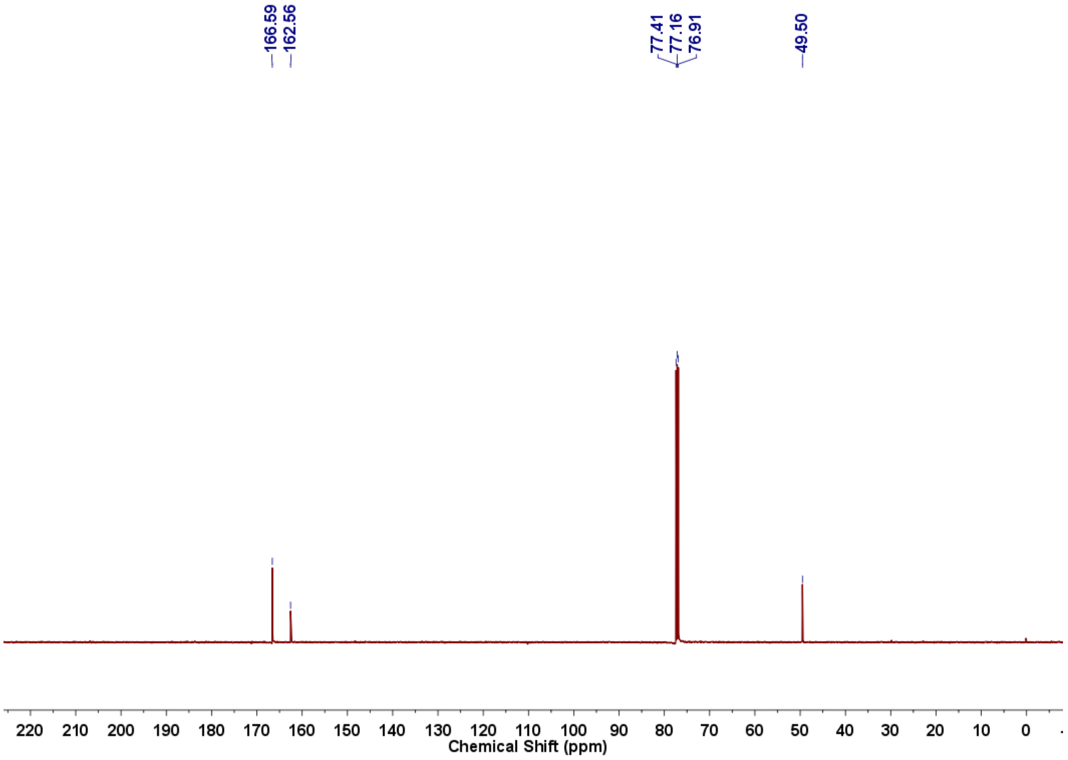

Figure S6. ^{13}C NMR spectrum of NTOM.

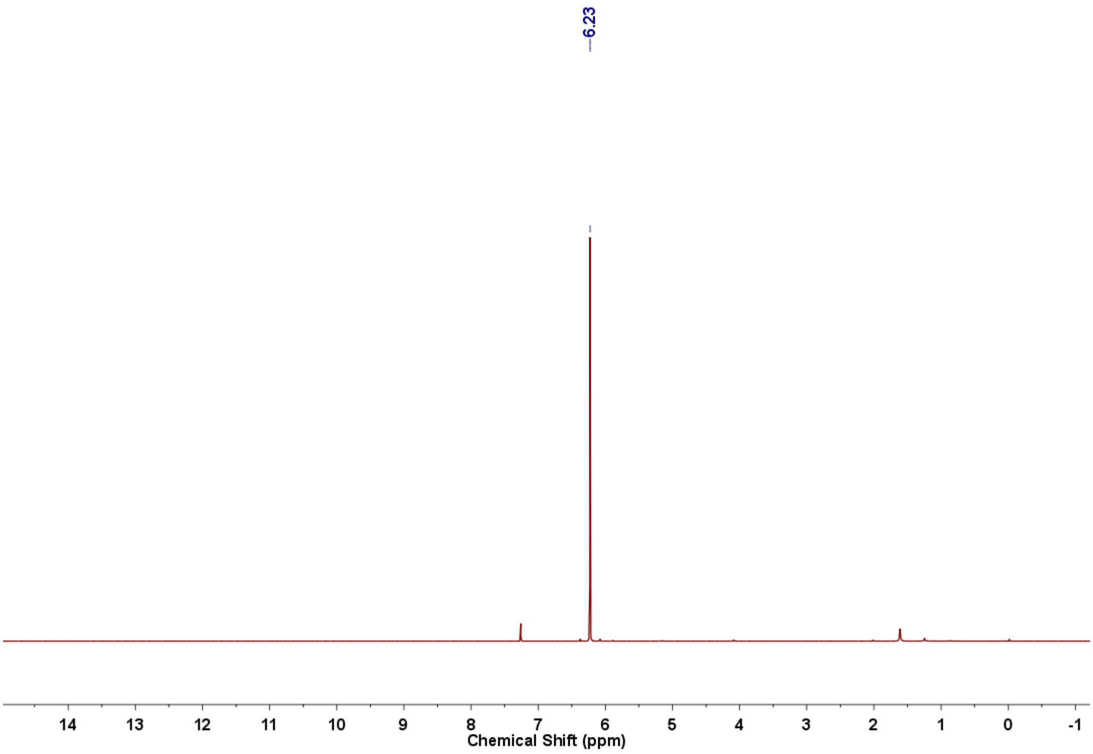

Figure S7. ^{1}H NMR spectrum of NTOF.

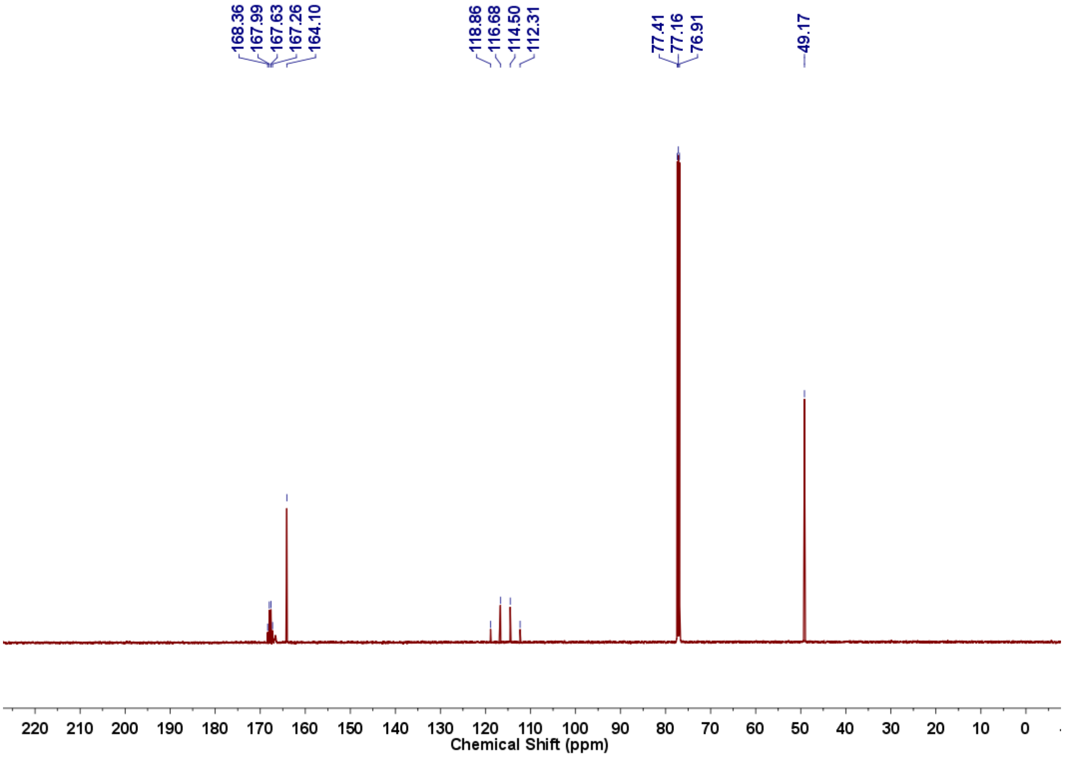

Figure S8. ^{13}C NMR spectrum of NTOF.

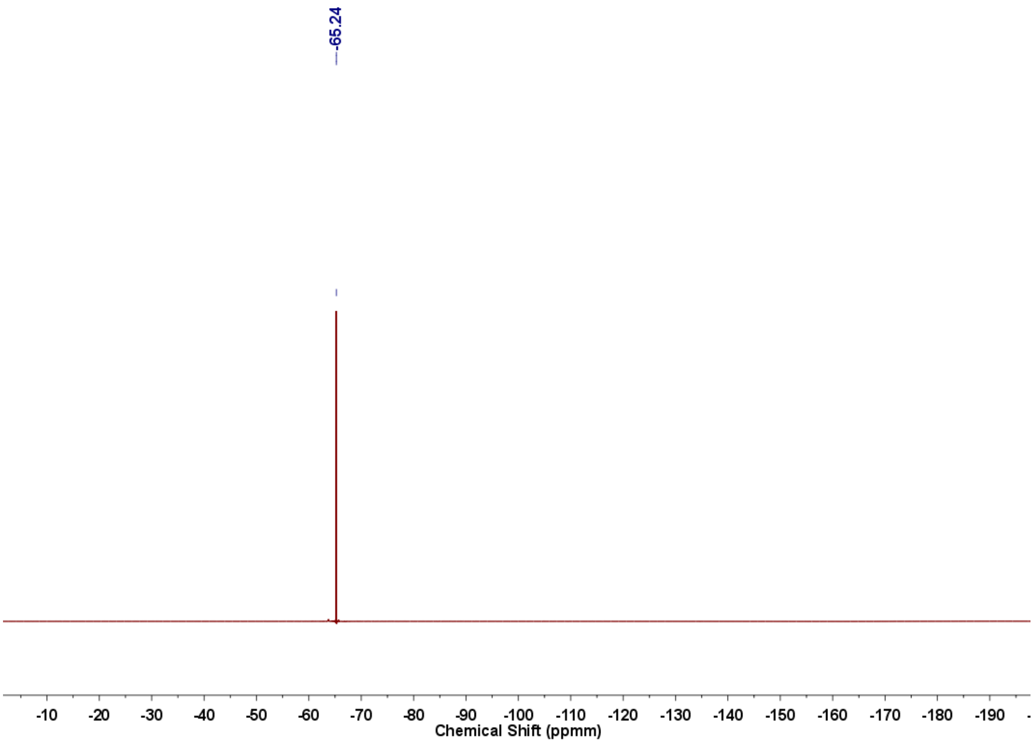

Figure S9. ^{19}F NMR spectrum of NTOF.

IRspectra

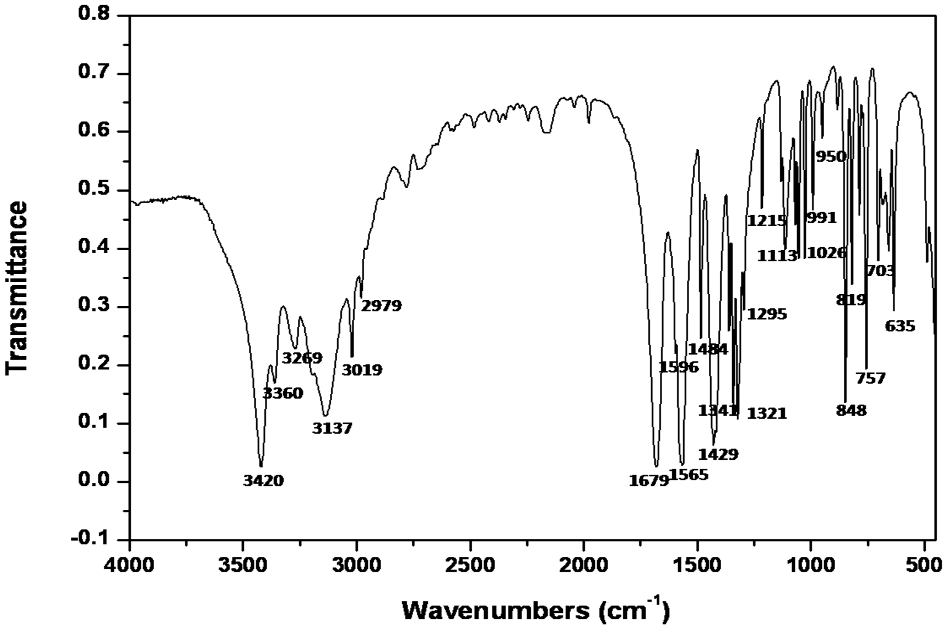

Figure S10.IR spectrum of NTOA

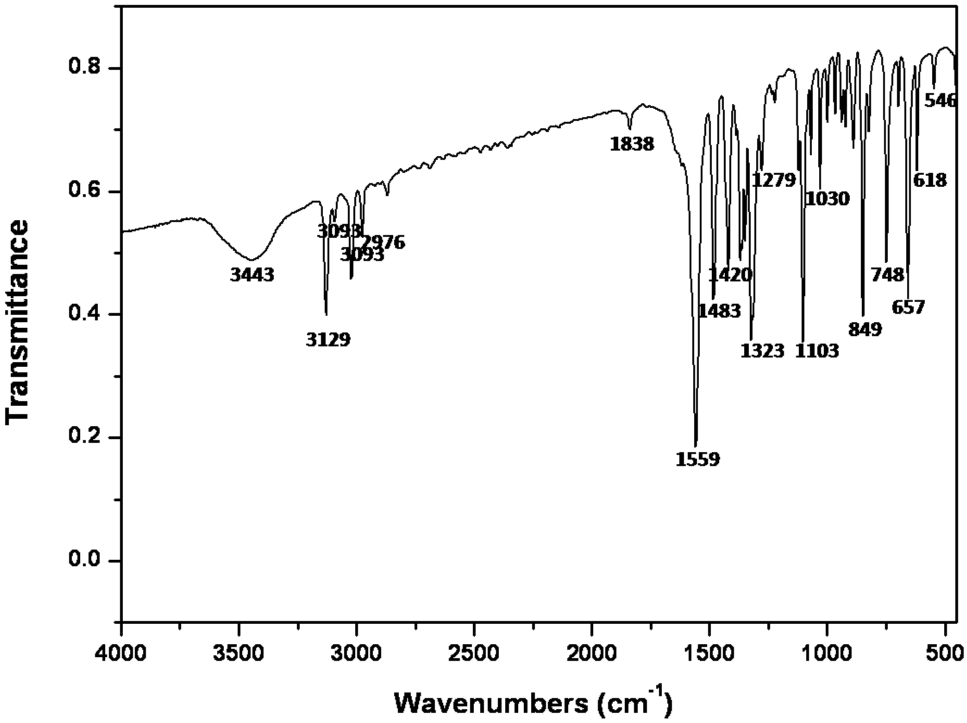

Figure S11. IR spectrum of NTOM.

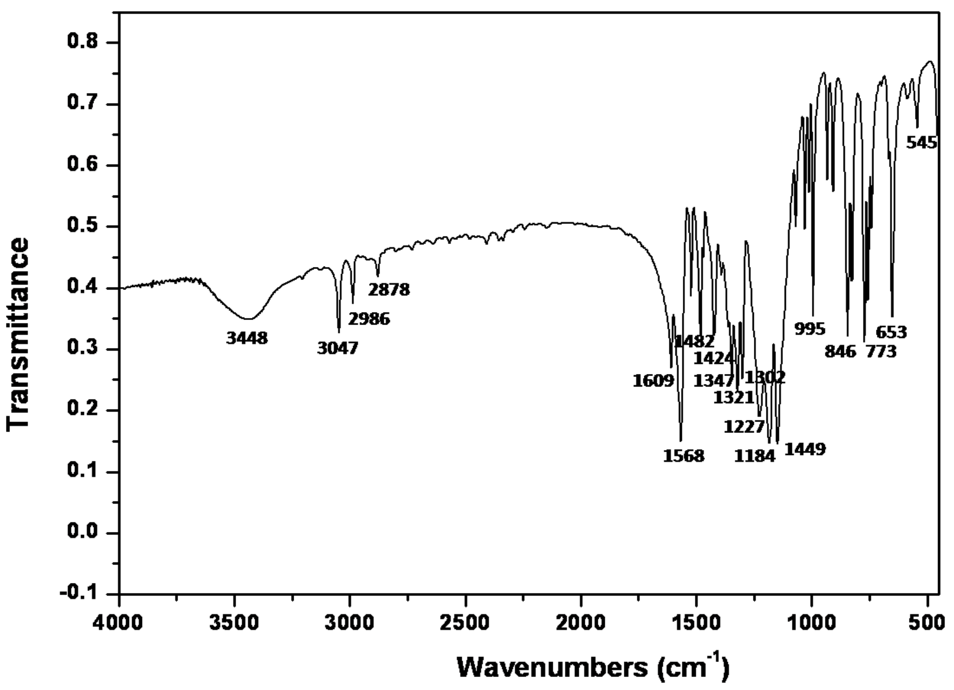

Figure S12. IR spectrum of NTOF.
